# Regulated competition in health care: Switching and barriers to switching in the Dutch health insurance system

**DOI:** 10.1186/1472-6963-11-95

**Published:** 2011-05-10

**Authors:** Margreet Reitsma-van Rooijen, Judith D de Jong, Mieke Rijken

**Affiliations:** 1 NIVEL-Netherlands Institute for Health Services Research, PO Box 1568, 3500 BN Utrecht, The Netherlands

## Abstract

**Background:**

In 2006, a number of changes in the Dutch health insurance system came into effect. In this new system mobility of insured is important. The idea is that insured switch insurers because they are not satisfied with quality of care and the premium of their insurance. As a result, insurers will in theory strive for a better balance between price and quality. The Dutch changes have caught the attention, internationally, of both policy makers and researchers. In our study we examined switching behaviour over three years (2007-2009). We tested if there are differences in the numbers of switchers between groups defined by socio-demographic and health characteristics and between the general population and people with chronic illness or disability. We also looked at reasons for (not-)switching and at perceived barriers to switching.

**Methods:**

Switching behaviour and reasons for (not-)switching were measured over three years (2007-2009) by sending postal questionnaires to members of the Dutch Health Care Consumer Panel and of the National Panel of people with Chronic illness or Disability. Data were available for each year and for each panel for at least 1896 respondents - a response of between 71% and 88%.

**Results:**

The percentages of switchers are low; 6% in 2007, 4% in 2008 and 3% in 2009. Younger and higher educated people switch more often than older and lower educated people and women switch more often than men. There is no difference in the percentage of switchers between the general population and people with chronic illness or disability. People with a bad self-perceived health, and chronically ill and disabled, perceive more barriers to switching than others.

**Conclusion:**

The percentages of switchers are comparable to the old system. Switching is not based on quality of care and thus it can be questioned whether it will lead to a better balance between price and quality. Although there is no difference in the frequency of switching among the chronically ill and disabled and people with a bad self-perceived health compared to others, they do perceive more barriers to switching. This suggests there are inequalities in the new system.

## Background

Since the 1990s the Dutch health care system has been in a process of transition from a mainly supply- orientated system towards a more demand-orientated system with managed competition. The general idea behind managed competition in health care is that it leads to better accessibility, higher quality and affordable prices in health care. Many other countries have introduced some form of managed competition in their health care system [1,2], for instance Germany, Switzerland, Belgium and the United States [3-6].

In the Netherlands a major step towards a demand-orientated system with managed competition was taken on 1 January 2006 by introducing a new health insurance law. In the old system of health insurance most people were publicly insured (about 67% of the Dutch population with an income below a certain maximum) and other people were privately insured. Insurance was not obliged for those who did not qualify for public insurance. In the new system of health insurance, there is a basic and a complementary insurance. The basic insurance is obligatory for everyone who lives in the Netherlands or pays tax on earnings in the Netherlands. Complementary insurance is not obligatory. The healthcare covered by the basic package includes among others, care by general practitioners and specialists, hospital care and medicines. The complementary insurance covers for example, dental care, manual therapy and glasses. Every year, during an annual enrolment period, every insured person can switch their insurer and/or their insurance plan. In this article we focus on switching from insurer.

This mobility of the insured is important in the new health care system. The idea is that if the insured switch and they do this because they are dissatisfied with the premium and quality of care of their insurer, this will force insurers to improve their balance between price and quality. Although there are many different quality aspects, such as level of service and coverage of the insurance package, the assumption of the health insurance system is that people switch because they are dissatisfied with their quality of care that is contracted by the health insurance company and premium. So the number of switchers, their characteristics and the reasons for switching are important in the new health insurance system.

The assumptions behind the new health insurance system are that people want to switch insurer and that the opportunity to switch is the same for everybody. The new system of health insurance is explicitly designed to create the opportunity to switch health insurer for all people and to the same extent. All insurers are obliged to accept everyone for the basic package. However, for complementary insurance, insurance companies can refuse applicants. Consequently, risk selection might play a role on the level of the complementary insurance. Although the insured are allowed to have their basic insurance with a different insurer than their complementary insurance, the possibility of risk selection through the complementary insurance might restrain the insured from switching. It is unlikely that the insured will only switch their basic insurance as it is (administratively) unattractive. If people have their basic insurance at another insurer than their complementary insurance, they have to register at two different insurers. If they have to declare health related costs, they will have to figure out what is covered by which of their insurances. In general, insurers discourage people from taking out their basic insurance with a different insurer. For example they will ask a higher premium for complementary insurance if they do not already have their basic insurance with the same insurer [7]. In order to decrease incentives for risk selection for the basic insurance, health care insurers share the risks related to certain health characteristics and age by a system of risk equalization [8]. There is financial compensation for insurers who accept insured people with a higher risk for the basic assurance. These may include the elderly, chronically ill and other people who have higher health costs. In this way, risk adjustment removes the financial incentive to select according to risks or to avoid insuring the sick [9]. Therefore, risk selection should not be a barrier to switching.

The international literature shows that, usually, few people switch health insurance [4]. In the old system in the Netherlands, approximately 3% of the publicly insured and 6% of the privately insured switched each year [10,11]. The percentage of switchers in the general population at the introduction of the new insurance system was rather high (21%) compared to the number of switchers in the old system [12,13].

An important question is whether the high number of switchers in 2006 was a unique event, or not. There are several reasons why in 2006 many people took the opportunity to switch insurance company. There was a lot of media attention given to the introduction of the new health insurance law compared to the years after, which might have led to a higher number of switchers in 2006. Furthermore, in the old system people with private insurance could not easily switch insurer because of barriers like risk selection and premium differentiation. In the new system switching was much easier for them. So, in 2006 many privately insured people may have taken the chance to switch. Last but not least, in the first year of the new system, insurance companies were more generous in accepting people for complementary insurance than in the years after [14].

Although there were reasons to expect that the number of switchers in the years after the introduction of the new system would not be as high as in 2006, we expected it to be higher than in the old system. Switching is easier than in the old system, especially for certain groups such as people with chronic illness or disability who had a private insurance under the old system.

The exact percentage of 'switchers' needed for the market to work as intended is difficult to determine. If too many people switch insurer this could also have negative effects. Collective costs will increase, which in turn could be the cause of increased premiums. Furthermore, insurance companies need the money from those who stay to accomplish desired improvements. However, it is also argued that the mere threat of switching is enough to force insurers to improve. Hirschman [15] distinguished two ways in which consumers can express their dissatisfaction with an organization: exit and voice. Voice means that people express their dissatisfaction, but that they do not leave the organization. For insurers, voice is important, as it provides them with information on what has to be improved while they can still use the money from the people who did not switch to accomplish these improvements. These insured people provide information on both quality of care and premium in order to reach the intended goals. However, it can be questioned whether people will ever use voice when switching is relatively easy. Voice probably works bests when groups of people complain and there is a threat of losing a large number of people, which can be the case in collectives. Collective insurance contracts are an interesting aspect of the new Dutch health insurance system. Any group of people, e.g. united through their work place, a sports association or patient organization, can take out insurance at a discount of a maximum of 10% on the basic insurance package if an insurance company is interested in offering a collective contract.

However, in order to make the system work it is not only important that people switch between health insurers, it is also important to know why they are switching. If people only switch because of the premium, they will not provide insurers with information on the quality of care. Consequently, insurers will then compete on the premium and not on the quality of care. Therefore, it is also interesting to look at the characteristics of switchers and the reasons for switching. In 2006 the quality of care was not an important reason for switching; the most reported reason for switching was a collective offer [12,13]. Collective offers are probably an important reason for switching because of the discount on the premium. Furthermore, choosing between collective offers makes the choice situation less complex since there are less insurance policies to choose from. Differences regarding quality of care are not likely for collective contracts through employers since employers mainly emphasise price of collective contracts. Most collective contracts are employer based. Patients' collectives, in particular, emphasise service and quality of care aspects of collective contracts. However, only a small percentage of people opt for these collective contracts. Differences in quality of care between collective contract is therefore not likely to be important in the decision of insured.

Are the reasons why people switch still a collective offer and lower premium, or do switchers also take quality aspects into account? The reasons for switching might differ between groups. People with chronic illness or disability have substantial experience with health care and are able to judge the level of service and the quality of care. Therefore these groups might base their choice to switch more often than the general population on these aspects. In addition, people with chronic illness or disability probably have more informed knowledge of what kind of care they may need for their illness and therefore know what they need insurance for. Therefore, the content of the insurance package is more important for them than it is for the general population.

Although the reasons for switching might differ between groups, the question is whether the numbers of switchers differ between groups. The new insurance system was explicitly designed to create equal opportunities to switch health insurer for all people and to the same extent. However, in 2006, people with chronic illness or disability switched health insurer less often than the general population [12]. This difference between the general population and people with chronic illness or disability could be explained by the fact that older people and lower educated people tend to switch less often and it is these groups who are over-represented among people with chronic illness or disability. These differences disappeared when corrected for age, sex, education and self-perceived health. Also empirical studies in several countries have shown that younger people, highly educated people and people in relatively good health, switch health insurer more often [12,16-21]. These differences in switching behaviour between these groups can be seen as a sign of the existence of inequalities and are often explained in terms of barriers. Older people, people with a relatively bad health and chronically ill and disabled people may perceive more barriers to switching, because these individuals want to have certainty about their continued coverage for health care [22,23]. Higher educated people might be more willing to take risks, and for them it will probably be easier to process all the relevant information on the available health insurers, making it easier for them to choose another health insurer [24]. Therefore, inequalities might not only become visible in differences in the numbers of switchers between different groups, but also in differences in the extent to which people perceive barriers to switching. If some people do perceive more barriers than others, this also might point towards inequalities in the new health insurance system.

In this article we focus on differences in switching between groups of insured people, the reasons for (not) switching and perceived barriers to switching. We examine whether there are differences in switching behaviour and the reasons for (not) switching between the general population and a specific group of insured, namely people with a chronic illness or disability. We also look at differences between groups defined in terms of socio-demographic and health characteristics. The main focus is on differences between groups over the three years 2007 to 2009.

In this article three questions will be answered:

1. How many insured people switched insurer in the years following the introduction of the new system (2007-2009) and are there differences between the population categories, defined in terms of socio-demographic and health characteristics?

2. What are the reasons for switching? Do switchers base their choice on the premium and the quality of care?

3. What are the reasons for not switching? Are there groups of people who perceive barriers more often than other groups?

### Hypotheses

In general, we hypothesize that people who are relatively frequent users of care for example, women [25] the elderly and lower educated people, switch less often than people who use health care less frequently. Therefore, we hypothesize that:

a In the general population people switch more often than people who are chronically ill and disabled

b Younger people switch more often than older people

c Higher educated people switch more often than lower educated people

d Healthier people switch more often than unhealthier people

e Men switch more often than women

In addition, we hypothesize that people with chronic illness or disability perceive more barriers to switching than the general population.

## Methods

We sent questionnaires to members of the Dutch Health Care Consumer Panel, a cross section of the Dutch population and the National Panel of people with Chronic illness or Disability (NPCD) in three consecutive years (2007, 2008 and 2009). The questionnaires were returned by 71% to 81% of the members of the Dutch Health Care Consumer Panel and by 82% to 88% of the members of the NPCD. In Table 1 an overview is given of the number of questionnaires that were sent and returned each year and of the response percentages. The protection of the collected data from both panels was laid down in privacy regulations, safeguarding ethical consent, and registered with the Dutch Data Protection Authority (nr. 1262949 and nr. 1283171).

**Table 1:** The number of questionnaires that were sent and returned and the response percentages for each year for the Dutch Health Care Consumer Panel and the National Panel of people with Chronic illness or Disability

	Dutch Health Care Consumer Panel			National Panel of people with Chronic illness or Disability		
	Sent	Returned	Response	Sent	Returned	Response
2007	2876	2325	81%	3761	3310	88%
2008	2796	2107	75%	3917	3290	84%
2009	2655	1896	71%	3509	2877	82%

### Dutch Health Care Consumer Panel

The Dutch Health Care Consumer Panel [26] consists of about 3000 people aged 18 years and older. The panel is representative of the general population for age and sex. Members of the panel receive a questionnaire four times a year and can quit the panel any time. Every two years, one third of the panel members is renewed. This renewal ensures that the panel remains a cross-section of the population, that members do not develop specific knowledge of, and attention for, health care issues, and that no "questionnaire-fatigue" occurs. New members of the panel are sampled from the general population. Sampled people receive an information letter about the panel and are called within a week after receiving that letter. If they are interested they receive first a questionnaire on background characteristics. When that questionnaire is returned they are considered members of the panel.

### National Panel of people with Chronic illness or Disability (NPCD)

The NPCD is a nationwide prospective panel-study in the Netherlands [27,28]. It has been set up to provide information about the consequences of chronic illness and disability from the patient's perspective. The panel consists of about 3500 people, aged 15 years and over with one or more medically diagnosed chronic disease(s) and/or moderate to severe levels of physical disability. The largest section of the panel's membership is recruited from general practices (see [27] for more details). Every year 500 new panel members with a somatic chronic disease are selected via a standardized procedure applied to a random sample of general practices throughout the country. This is done in order to replace panel members who have dropped out or who have participated for the maximum term of four years. Additional panel members are selected from several national population surveys on the basis of the presence of a moderate to severe physical disability. Because of this procedure, the NPCD can be considered a representative sample of the Dutch population of adult, non-institutionalized (physically) chronically ill and/or disabled persons.

### Measurements

Among other things, we asked whether they switched insurer (0 = did not switch, 1 = switched). Subsequently, we asked switchers for the reasons for switching and people who did not switch for the reasons for not switching. In a list of reasons for not switching respondents could indicate which reasons applied to them (0 = do not apply, 1 = apply). Among this list were four possible barriers people could perceive to switching. These were: ' I just couldn't be bothered to look for another better or cheaper health care insurer'; 'It is impossible for me to change to another health care insurer, I think'; 'I am afraid they will not accept me for a complementary insurance'; and 'I fear getting into (administrative) problems if I change to another health insurance company'. These barriers were chosen using responses in previous years.

### Analyses

#### Composition of the two panels

The panels used for this article are not comparable on age, sex and education (Table 2). The composition of the Dutch Health Care Consumer Panel is more younger, higher educated, and male, compared to the NPCD panel. This pattern is consistent over the three years. It might be that possible differences in switching behaviour between the general population and people with chronic illness or disability are not caused by chronic illness or disability, but that they are caused by differences in composition of the two panels with regard to age, sex and education. To make both panels comparable on these three characteristics, a weight factor for people with chronic illness or disability was used. People who had one or more missing values on these characteristics were removed, because weight factors based on these characteristics could not be estimated (in total 2,8% of the respondents were removed). We only analysed results from people who were at least 18 years old, since people below 18 years are not insured by themselves. One variable with eighteen weight factors was used for the combination of age (18-44; 45-65; 65 and older), sex (male; female) and education (lower; intermediate; higher). To disentangle the differences in switching behaviour that are caused by differences in socio-demographic composition and by chronic illness or disability, we present both the unweighted and the weighted percentages.

**Table 2:** Description of respondents: age, sex and education in percentages

	General population			People with chronic illness or disability		
	2007	2008	2009	2007	2008	2009
						
**Age**						
18-44	25	23	20	12	10	9
45-64	48	48	49	42	41	42
≥ 65	27	28	31	47	48	49
**Sex**						
Male	44	43	45	36	36	39
**Education**						
Lower	22	22	22	46	45	42
Intermediate	48	47	47	39	39	40
Higher	31	31	31	15	16	18

The description of respondents in percentages and of the percentages of switchers, and reasons for not switching, are standardized to a standard population based on the proportion between the number of chronically ill people and the number of disabled people in the Dutch population, by using a weight factor.

#### Testing for differences between groups

To test for differences between groups, we performed logistic regression analyses and took into account the differences in socio-demographic characteristics between both panels by including age, sex, education and self-perceived health status as predictors. For all analyses we used the statistical program STATA version 10.1. We took into account the dependency of our data (panel members can be a respondent in more than one year) by clustering on the unique personal panel identification number.

## Results

### Switching health insurer

In the general population 6% switched insurer in 2007, 4% in 2008 and 3% in 2009 (Table 3). The weighted percentages for people with chronic illness or disability are in all three years the same as the percentages of switchers in the general population. Unweighted percentages show that in 2007, 2008 and 2009 respectively 5%, 4% and 2% of people with chronic illness or disability switched insurer. These percentages of switchers are remarkably lower than the percentages of switchers on the introduction of the health insurance reform in 2006. In that year 21% of the adult population switched health insurer [12,13]. The percentage of switchers in 2007-2009 is comparable to the percentage of switchers in the situation before the health insurance reform, when it was approximately 3% for the publicly insured and 6% for the privately insured [10,11].

**Table 3:** Percentage of people switching health insurer and their characteristics, weighted for age, sex, and education, unweighted percentages between brackets

	General population			Chronically ill and disabled*		
	2007	2008	2009	2007	2008	2009
	N = 2232	N = 2047	N = 1853	N = 3144	N = 3119	N = 2761
Percentage of switchers	6	4	3	6 (5)	4 (4)	3 (2)

We performed logistic regression analyses to test for differences in the numbers of switchers between population categories, defined in socio-demographic and health characteristics. We took into account the dependency of our data by clustering on the unique personal panel identification number. The results (see Table 4) show that older people switch less often than younger people. Women switch more often than men. Higher educated people switch more often than lower educated people. People with a good self-perceived health status do not switch more often than people with a bad self-perceived health status. The percentage of switchers in 2008 and 2009 is significantly lower than in 2007. There is no difference between the number of switchers in the the general population and people with chronic illness or disability.

**Table 4:** Logistic regression model for switching health insurer (N = 14897)

	Odds Ratio	p-value
**Age**		
18-44	Reference	
45-64	0.63	0.000
≥ 65	0.47	0.000
**Sex**		
Male	Reference	
Female	1.52	0.000
**Education**		
Lower	Reference	
Intermediate	1.25	0.054
Higher	1.42	0.007
**Self-perceived health status**		
Bad	0.83	0.178
Good	0.89	0.336
Very good	Reference	
		
**Group**		
Dutch Health Care Consumer Panel	0.86	0.143
Chronically ill and Disabled	Reference	
		
**Year**		
2007	Reference	
2008	0.66	0.000
2009	0.45	0.000

Although there are no differences between the numbers of switchers in the general population and people with chronic illness or disability, there might be differences in the reasons for not switching between the groups. Therefore, we asked the switchers for their reasons for switching and the people who did not switch for their reasons for not switching.

### Reasons for switching health insurer

We examined which reasons are reported for switching and if there are differences between people with chronic illness or disability on the one hand and the general population on the other hand. As the number of switchers is very low, we cannot perform reliable analyses on reasons for switching to test for differences between groups. However, it is interesting to examine what reasons for switching are most often reported, and whether reported reasons are consistent over time. Both for the general population and for people with chronic illness or disability, we looked at the top three most reported reasons for switching for each year. The top three most reported reasons is highly comparable for both groups and is comparable over the years. For both groups dissatisfaction with the premium of the package offered and a collective offer are in the top three of the most reported reasons in all three years. There was one exception, namely the collective offer is not in the top three in 2008 for people with chronic illness or disability. In two of the three years (2008 and 2009) dissatisfaction with the coverage of the complementary insurance is one of the three most reported reasons for both groups. Other top three reasons for people with chronic illness or disability are dissatisfaction with the coverage of the package offered (2007) and dissatisfaction with the service of the insurer (2008). Another top three reason for the general population was dissatisfaction with the premium of the collective offer (2007). Considerations of the quality of care did not seem to play a role in switching behaviour.

### Reasons for not switching health insurer

We also asked people who did not switch for their reasons for not switching. The reasons for not switching were only asked for both panels in 2007 and 2008. Just as was the case with the top three of the reasons for switching, the top three most reported reasons for not switching is highly comparable for both groups and over the years. In both years and both groups, 'I have been with this health insurance company for such a long time already', and 'I'm satisfied with the coverage of the package offered' are two of the most reported reasons. For the general population the reason, 'I'm satisfied with the service of my health insurer', is in both years in the top three and for the chonically ill and disabled, 'I'm satisfied with the coverage of the complementary insurance', is in both years in the top three.

Respondents could also report if they perceived barriers to switching. In total we presented four barriers to switching and respondents could indicate if these barriers were a reason for them for not switching. In table 5 the four different barriers are presented as well as the percentage of the general population and that of people with chronic illness or disability that perceived these barriers each year.

**Table 5:** Perceived barriers to switching, unweighted percentages between brackets

	General population		Chronically ill and disabled	
	2007	2008	2007	2008
	N = 2112	N = 1971	N = 2957	N = 2984
1. I just couldn't be bothered to look for another, better or cheaper health care insurer	15	19	19 (17)	17 (15)
2. It is impossible for me to change to another health care insurer, I think.	2	4	8 (9)	8 (8)
3. I am afraid they will not accept me for a complementary insurance.	5	6	14 (12)	16 (14)
4. I fear getting into (administrative) problems if I change to another health insurance company.	6	5	11 (11)	10 (10)

To test whether some groups of people, defined in socio-demographic and health characteristics, perceive these barriers more often than other groups of people, we performed logistic regression analyses. We again clustered on the unique personal panel identification number. The results show (see table 6) that overall people with a bad self-perceived health status and chronically ill and disabled people perceive more barriers than people with a good self-perceived health status and people in the general population.

**Table 6:** Logistic regression model for perceived barriers to switching health insurer (N = 9880)

	Could not be bothered		Impossible for me to change		Afraid they will not accept me		Fear getting into (administrative) problems	
	Odds Ratio	p-value	Odds Ratio	p-value	Odds Ratio	p-value	Odds Ratio	p-value
**Age**								
18-44	reference		reference		reference		reference	
45-64	0.75	0.001	1.25	0.205	1.29	0.045	.93	0.591
≥ 65	0.67	0.000	1.14	0.460	0.74	0.030	.86	0.284
**Sex**								
Male	reference		reference		reference		reference	
Female	1.08	0.228	.94	0.534	0.82	0.017	.91	0.301
**Education**								
Lower	reference		reference		reference		reference	
Intermediate	1.39	0.000	.89	0.288	1.20	0.050	1.14	0.172
Higher	2.21	0.000	.88	0.361	1.29	0.032	1.29	0.033
**Self-perceived health status**								
Bad	1.21	0.042	4.57	0.000	5.47	0.000	2.54	0.000
Good	1.10	0.255	1.91	0.003	2.12	0.000	1.54	0.002
Very good	reference		reference		reference		reference	
**Group**								
Dutch Health Care Consumer Panel	0.91	0.159	0.49	0.000	0.51	0.000	0.68	0.000
Chronicall-y ill and Disabled	reference		reference		reference		reference	
								
**Year**								
2007	Reference		Reference		Reference		reference	
2008	1.02	0.611	1.16	0.068	1.24	0.000	0.87	0.025

## Discussion

We have studied the switching behaviour of insured people and the reasons for not switching over three consecutive years (2007, 2008 and 2009), following the introduction of the new health insurance system.

### Numbers of switchers

First we looked at the numbers of switchers in these three years and at differences in numbers of switchers between population categories, defined in socio-demographic and health characteristics. The numbers of switchers were in all three years low compared to 2006 and comparable to the numbers of switchers in the old health insurance system. Our general hypothesis that people who are relatively frequent users of care switch less often than people who are less frequent users of care is partly confirmed. Older people and lower educated people switch less often than others. We hypothesized a difference between people with chronic illness or disability and the general population. However, this hypothesis was not confirmed in our research. The fact that we did not find a difference in switching behaviour between these two groups is comparable to a similar study on switching behaviour in 2006 [12]. In that study it was also found that there was no difference in switching behaviour between these groups when controlled for age, education, and sex. We did not find a difference in switching behaviour between people with a bad, compared to a good, self-perceived health status. Obviously, the opportunity to switch health insurer is the same for these groups, which is one of the goals of the new system. We expected that women would switch less often than men, but found the reverse. The results from other studies concerning the relationship between switching behaviour and gender are inconsistent [17,19].

### Reasons for switching

Second, we examined the reasons for switching. The most reported reasons for switching were a collective offer and dissatisfaction with the premium of the package offered, dissatisfaction with the coverage of the complementary insurance, dissatisfaction with the coverage of the package offered, dissatisfaction with the service of the insurer and dissatisfaction with the premium of the collective offer. The quality of care did not seem to play an important role in switching behaviour.

### Reasons for not switching

Third, we looked at reasons for not switching and barriers to switching. Furthermore, we examined if some population categories perceived more barriers to switching than others. The most reported reasons for not switching were: 'I'm insured with the same insurer for years' and; 'I'm satisfied with the coverage of the package offered'; 'I'm satisfied with the service of my health insurer' and; 'The coverage of the complementary insurance'. More important, our hypothesis that chronically ill and disabled, and people with a bad self-perceived health status perceived more barriers to switching than the general population and people with a good self-perceived health status, has been confirmed.

What do our results mean for the success of the new health insurance system? One of the goals of the new health insurance system was to stimulate the mobility of the insured. If insured people switch they should ideally choose the best insurer, in terms of quality and premium. This should stimulate insurers to buy good care at a good price. But is about 5% of the insured switching each year enough? If too many people switch insurer, this could also have negative effects. Collective costs will increase, which in turn could be the cause of increased premiums. In any case insurance companies need the money from those who stay to accomplish desired improvements.

The answer to the question of whether 5% switchers is enough to reach the intended goals is also related to the reasons that are reported for switching. For the intended functioning of the new health insurance system it is important that a part of the switchers chooses another insurer because of the quality and premium of their current insurer. But our results show that the quality offered by the insurer is not the main reason for switching. The premium and a collective offer are more important. So, it might be questioned whether the signals that are provided by the switchers are enough to stimulate the insurers to strive for a better balance between price and quality. Collectives could play a role, since they negotiate with insurance companies. However, other studies show that for the most sizeable collectives, namely employers, the premium is most important. Quality is less important in the negotiations [29]. For individuals, collectives make choosing an insurer easier, since collectives reduce the choice of options: The number of insurers to choose from is much larger than the number of collectives.

The fact that people do not mention quality of care as a reason for switching is consistent with the finding that people do not recognise differences between health insurers with regard to their quality of care. The performance of health plans is assessed annually in the Netherlands using the standardized CQI^® ^health plan instrument 'Experiences with Health care and Health Insurer'. Consumer Quality Index (CQ-index or CQI) instruments assess the quality of health care seen from the consumer's perspective, and measure consumers' experiences instead of inquiring about their satisfaction [30-36]. These studies show that, from the insured persons' perspective, there is almost no difference between insurers in their quality of care.

The new insurance system was explicitly designed to create equal opportunities to switch health insurer for all people - and to the same extent. Still, some groups could benefit more from market competition than others, like younger and higher educated people. Although we did not find a difference in switching behaviour between the general population and people with chronic illness or disability, chronically ill and disabled perceive more barriers than others. The same was found when we compared people with a bad self-perceived health status to people with a good self-perceived health status. Although perceived barriers might not exist in practice, perceived barriers are, in their effect, real barriers since these barriers prevent certain groups of people from switching. So, although the system is explicitly designed to create equal opportunities to switch health insurer, some groups of people do switch more often than others and some groups of people do perceive more barriers than others. These findings point towards the existence of inequalities in the new health insurance system. The fact that some groups of people are more afraid of not being accepted, or think that it is not possible for them to have a complementary insurance, might be interpreted as self-selection. Therefore the role of perceived barriers in switching behavior might play a bigger role in the future and might lead to inequalities in switching behavior. It is important to monitor this process, especially when differences between insurers will occur.

## Conclusion

The percentages of switchers in 2007-2009 were considerably lower than the percentage of switchers in 2006 and are comparable to the number of switchers in the old system. Switching is not based on quality considerations. The question is whether the mobility of the insured in the new health insurance system will lead to insurers improving the quality of care offered. Although the chronically ill and disabled and people with a bad self-perceived health did not differ in the frequency of switching compared to others, they perceived more barriers to switching. These findings point to the existence of inequalities in perceived possibilities for switching in the new health insurance system.

## Competing interests

The authors declare that they have no competing interests.

## Authors' contributions

MRvR drafted the manuscript and performed the statistical analyses. JdJ and MR contributed to the acquisition of the data and were involved in drafting the manuscript and critical revision of this manuscript. All authors have given final approval of the submitted manuscript.

## Pre-publication history

The pre-publication history for this paper can be accessed here:

http://www.biomedcentral.com/1472-6963/11/95/prepub
